# Snake venom bioprospecting as an approach to finding potential anti-glioblastoma molecules

**DOI:** 10.1590/1678-9199-JVATITD-2024-0015

**Published:** 2024-09-16

**Authors:** Javier Orozco-Mera, Alejandro Montoya‐Gómez, Daiana Silva Lopes, Eliécer Jiménez‐Charris

**Affiliations:** 1 Grupo de Nutrición, Facultad de Salud, Universidad del Valle, Cali, Colombia.; 2 Department of Neurosurgery, Clínica Imbanaco, Cali, Colombia; 3 Multidisciplinary Institute in Health, Federal University of Bahia (UFBA), Vitória da Conquista, BA, Brazil.

**Keywords:** Bioprospecting, Viperidae, Snake venom, Drug discovery, Glioblastoma, Therapeutics

## Abstract

Glioblastoma (GB) is the most common type of malignant tumor of the central nervous system, responsible for significant morbidity and with a 5-year overall relative survival of only 6.8%. Without advances in treatment in the last twenty years, the standard of care continues to be maximum safe resection, Temozolomide (TMZ), and radiotherapy. Many new trials are ongoing, and despite showing increased progression-free survival, these trials did not improve overall survival. They did not consider the adverse effects of these therapies. Therefore, an increasing number of bioprospecting studies have used snake venom molecules to search for new strategies to attack GB selectively without producing side effects. The present review aims to describe GB characteristics and current and new approaches for treatment considering their side effects. Besides, we focused on the antitumoral activity of snake venom proteins from the Viperidae family against GB, exploring the potential for drug design based on *in vitro* and *in vivo* studies. This review followed the Preferred Reporting Items for Systematic Reviews and Meta-Analysis (PRISMA) guidelines. In January 2024, a systematic search was performed in the PubMed, EMBASE, and Web of Science databases from January 2000 to December 2023. Search terms were selected based on the population/exposure/outcome (PEO) framework and combined using Boolean operators ("AND", "OR"). The search strategy used these terms: glioblastoma, glioma, high-grade glioma, WHO IV glioma, brain cancer, snake venom, Viperidae, and bioprospection. We identified 10 *in vivo* and *in vitro* studies with whole and isolated proteins from Viperidae venom that could have antitumor activity against glioblastoma. Studies in bioprospecting exploring the advantage of snake venom proteins against GB deserve to be investigated due to their high specificity, small size, inherent bioactivity, and few side effects to cross the blood-brain barrier (BBB) to reach the tumor microenvironment.

## Background

The World Health Organization defines cancer as "a large group of diseases that can start in almost any organ or tissue of the body when abnormal cells grow uncontrollably, go beyond their usual boundaries to invade adjoining parts of the body, and spread to other organs" [1]. According to the GLOBOCAN report, it was estimated that in 2020, there were 19.3 million new cases per year and almost 10 million deaths [2]. GB is the second most frequent primary brain tumor and the first for malignant tumors, accounting for 48.6% of all malignant brain tumors [3]. It is characterized by its invasiveness, aggressiveness, recurrence, poor treatment response, and high mortality rate. Various treatments are currently used to manage GB, including the maximum safe resection followed by chemotherapy and radiotherapy. However, these therapies are not specific, so they are associated with many undesirable systemic effects for the patient. Therefore, new pharmacological alternatives from natural sources focusing on the tumor microenvironment and reducing side effects are required.

Thus, bioprospecting studies to find products with medical applications have considerably increased during the last decade, including drugs such as anticancer agents [4]. Snake venoms are valuable in bioprospecting since they contain proteins and peptides with antiplatelet, antiangiogenic, and antiproliferative effects [5]. Among the snake venoms, the most outstanding in terms of the search for new pharmacological molecules come from the Elapidae and Viperidae families. Moreover, most snake venom-based drugs approved for clinical use come from the Viperidae family [6]. For this reason, in this review, we focus on research based on molecules from the Viperidae family with anticancer effects on GB.

In this review, we first show an overview of GB. Next, we discuss current GB treatments, their patient's adverse effects, and the role of snake venoms in searching for new molecules for its treatment. Later, we focus on the published works regarding the venom from the Viperidae family and their results in searching for GB treating strategies, showing the *in vitro* and *in vivo* studies. In addition, we show the potential of isolated snake venom molecules to cross the blood-brain barrier. Finally, we conclude that snake venoms from the Viperidae family could provide an alternative to selectively attack GB since some of its molecules have demonstrated the ability to affect characteristics inherent to the progression of this type of tumor.

## Glioblastoma 

GB is the most frequent malignant tumor in the central nervous system. It progresses through different genetic pathways affecting patients of different ages, but it appears mainly in adulthood without gender or ethnicity predilection [7]. It is one of the most aggressive solid tumors and remains an incurable disease, characterized by its significant heterogeneity and multiple escape routes to treatment, with a 5-year overall relative survival of only 6.8% [7]. GB is classified into two types of tumors: primary and secondary GB. Primary GB is the most frequent (80%) and is present mainly in people over 60 years, with survival ranges between 12 and 15 months. This kind of tumor is characterized by the absence of mutations in Isocitrate dehydrogenase (IDH) and is defined as wild-type [8]. 

On the other hand, secondary GB has mutations in IDH 1 or 2. It appears in younger patients, and their survival can even reach 30 months, which carries a better prognosis than primary GB [8]. The tumor microenvironment (TME) is also crucial in GB progression. GB is characterized by infiltration of tumor-associated microglia and macrophages (TAMs) that are immune cells in the TME of GB, inducing immunosuppression and releasing mediators such as cytokines and growth factors, essentials for proliferation [9, 10]. In addition, TAMs promote glioblastoma angiogenesis mainly mediated by vascular endothelial growth factor (VEGF) and depletion of microglia-reduced tumoral vessels, which is crucial for metastasis [11]. Thus, clinical outcomes for this disease remain poor, and new treatment strategies are required.

## GB therapeutic strategies

### Classic therapies

The current treatment for GB consists of the maximum safe resection followed by chemotherapy and radiotherapy, known as the Stupp protocol [12]. Regarding surgery, many tools are used to maximize resection and preserve the functionality of patients, e.g., intraoperative cortical monitoring, image-guided surgery such as neuronavigation, or different fluorescent media for better visualization of the tumor in the surgical field. Partial or incomplete resection of the lesion relieves intracranial hypertension, decreases the mass effect, decreases dependence on steroids, increases the quality of life, reduces symptoms such as headache, and sometimes allows better control of seizures [13]. However, there are two significant problems: the first is the eloquent areas in the tumor vicinity, which often limits the complete resection of the lesion, and the second is that even when macroscopically complete resection is achieved, there are still areas where there is already infiltration or tumor cells migration that sometimes not even resonance studies can reveal and that later become foci of tumor regrowth. 

Regarding chemotherapy, the first line consists of TMZ. This alkylating agent is administered orally with radiotherapy at a dose of 75 mg/m^2^ during the 42 days of radiotherapy and subsequently in five-day cycles at a dose of 150mg/m^2^ followed by 23 days off, i.e., 28-day cycles for a minimum of six months [14]. Its adverse effects include thrombocytopenia, neutropenia, leukopenia, anemia, impaired liver function, nausea, vomiting, anorexia, constipation, diarrhea, headache, fatigue, and dizziness [15]. 

On the other hand, the standard radiotherapy used is divided into doses of 2 Gy per day for a cumulative dose of 60 Gy in six weeks. This dose may vary depending on the patient's condition or fractionation [16]. Between treatment adverse effects, we can mention long-term neurocognitive alterations, fatigue, nausea, vomiting, changes in skin color, alopecia, and headache, among others [17]. In addition, there is the risk of another brain tumor (in the long term) and radionecrosis. This last effect is a crucial diagnostic dilemma since magnetic resonance imaging, or computed tomography, resembles tumor relapse [18]. Furthermore, it can generate symptoms of intracranial hypertension, requires medical management, and may even occasionally require surgical intervention [7]. Tumor recurrence management is even more complicated since no consistent evidence of response to any treatment exists. Surgical reintervention can be contemplated as the first line of treatment, according to the patient's functional status. The second line of oncological management generally involves antiangiogenic agents such as Bevacizumab (BVZ) [7]. Finally, reintervention with radiotherapy is limited by the short time between the radiation and tumor relapse. Unfortunately, some patients relapse early, while others around the first 10 to 12 months, but relapse invariably appears [7]. 

### Emerging therapies

With new cancer therapies like immunotherapy or target therapy, GB has been the subject of many clinical trials with different approaches, such as checkpoint inhibitors, chimeric receptors on T cells, vaccines, viral therapy, or monoclonal antibodies [13]. Generally, the tumors that respond to immunotherapy have a high mutational load. However, despite their heterogeneity, the mutational load of GB is low. In addition, the complications associated with the inflammation generated by immunotherapy could complicate the clinical scenario of a patient with intracranial hypertension due to their tumor [7]. However, numerous trials have been carried out in this field, among which are worth mentioning:

### Checkpoint inhibitors

This therapy is based on the CTLA-4 (Cytotoxic T-Lymphocyte Antigen 4) and PD-1 (Programmed cell death protein 1) union of T lymphocyte receptors to their respective ligands CD80 (Cluster of differentiation 80) and PD-L1/2 (Programmed Death-ligand 1/2), generating attenuation of the immune response. Unfortunately, these receptors are not overexpressed in GB, which decreases the treatment response. Several phases II and III clinical trials have tested the use of PD-1 inhibitors such as Nivolumab, Pembrolizumab, and CTLA-4 inhibitors (Ipilimumab, alone or in combination with standard therapy (TMZ + radiotherapy)), with some changes in progression-free survival (PFS), without differences in overall survival (OS) [19, 20, 21]. 

### Chimeric receptors on T cells

Treating T cells with chimeric antigen receptors (CAR-T) has been used to amplify the immune response and generate tumor cytotoxicity. In GB, it has been treated with chimeric receptors against the epidermal growth factor receptor variant III (EGFRvIII), a mutation induced by the deletion of exons 2-7 [22]. It affects the extracellular receptor domain, causing its constitutive activation. This mutation is present in at least 50% of GB. A decrease in EGFRvIII expression has been shown in phase I trials after CAR-T therapy [22].

### Vaccines

Peptide and dendritic vaccines have been used. One of the most expected vaccines was Rindopepimut, directed against EGFRvIII. However, despite its good results in Phase I, it failed to pass the Phase III evaluation [23]. Later, another vaccine (SurVaxM) was used against Survivin, a member of the apoptosis inhibitors family, with good results in a phase II trial [7].

### Viral therapy

Therapy with viral agents in GB has been investigated using an experimental combination drug (Vocimagene amiretrorepvec) that includes a gene therapy agent and a prodrug. This combination drug, also known as Toca511, contains a gene that codes for Cytosine Deaminase (Toca FC); this enzyme degrades Flucytosine to 5 Fluoracil in the tumor once it has overcome its most crucial obstacle, the blood-brain barrier. Unfortunately, Toca511 has not shown any benefit in phase III trials despite the promising results obtained in the initial stages [24]. Oncolytic viruses have also been used, such as PVSRIPO, a chimeric polio-rhinovirus that recognizes the poliovirus receptor CD155 in tumor cells, with a great response in phase I trials, currently in phase II [7].

### Target therapies

Finding a targeted therapy in a tumor characterized by great intra- and intertumoral heterogeneity is complex. However, attempts have been made to exploit some pathways in many tumors, such as RAS/PI3k (Rat sarcoma virus protein/ Phosphoinositide 3-kinases), P53, and Retinoblastoma (Rb). On the other hand, there has been significant interest in EGFRvIII due to the use of tyrosine kinase inhibitors in other tumors with a substantial response, which has yet to occur in GB. Other targets of interest are BRAFV600E (v-raf murine sarcoma viral oncogene homolog B1), NTRK (neurotrophic tyrosine kinase-1 receptor), and CDK (Cyclin-dependent kinases) 4/6 kinase-dependent cyclins, which have similar results. Multi-target therapy is another approach. For example, Regorafenib, a multikinase inhibitor, is currently in clinical phase II in both *de novo* GB and recurrent GB. The main problem with these multiple-target inhibitors is their high toxicity due to drug-drug interactions, distinct pharmacokinetics, solubility, and bioavailability; some observed effects have been cardiovascular disease, colitis, or ileitis among others [15, 25]. 

### Monoclonal antibodies

This treatment modality has been directed against the ligands or the receptors. For example, BVZ is a monoclonal antibody directed against the VEGF that prevents binding to its receptor. This antibody showed an increase in survival-free progression, although without changes in overall survival. It has FDA approval for managing recurrent GB. On the other hand, Cetuximab is a monoclonal antibody directed against the epidermal growth factor receptor (EGFR); the EGFR gene amplification and protein overexpression in glioblastoma implies most aggressive to the tumor. Although bevacizumab has been recently approved for use as a single agent for patients with GBM, with progressive disease, most of the monoclonal antibody therapies have not translated into significant survival advantages and failed in phase II trials due to their ineffectiveness [15]. 

### Tumor-associated microglia and macrophages strategy target

Recently, TAMs represented a promising treatment strategy target for GB. Although historically, this tumor has been considered "immunologically cold," the TME of GBM can contain more than 30% of TAMs. These TAMs promote glioma cell proliferation and invasion, favor angiogenesis, and generate more immunosuppressive TME. In this scenario, multiple strategies have been used to make this TME a therapeutic objective, such as depletion of TAMs, reprogramming, enhancing phagocytosis, or reducing recruitment of TAMs. Phase I/II clinical trials are in progress using different molecules. For example, Emactuzumab (RG7155), a therapeutic anti-CSF-1R antibody, has been combined with the programmed cell death-1 ligand (PD-L1)-blocking mAb atezolizumab, Plerixafor (AMD3100) a CXCR4 antagonist or WP1066 a STAT3 inhibitor [26]. However, despite the arduous efforts in the search for new glioblastoma treatments, the agents tested as alternative therapies have yet to be shown to outperform the gold-standard treatment, temozolamide. Therefore, the search for new pharmacological alternatives continues, and snake venoms could be a new option.

### Snake venoms as pharmacological molecule sources

Snake venoms typically consist of a mixture of components, such as peptides and proteins. The principal protein families of the Viperidae snake venom are phospholipase A_2_ (PLA_2_), metalloproteinase, and serine protease, followed by minor components such as L-amino acid oxidase, C-type lectin-like proteins, disintegrins, cysteine-rich secretory protein, natriuretic peptides, and defensins. Envenomation with whole snake venoms can result in several adverse effects, including neurotoxicity, haemotoxicity, and cytotoxicity, depending on the snake species. In addition, it can produce acute skeletal muscle necrosis, flaccid paralysis, local inflammatory reactions, cell death induction, and platelet aggregation inhibition [27]. However, identifying protein sequences isolated from the Viperidae family's venoms has been used to develop pharmacological agents approved for clinical use [6]. 

For example, the antihypertensive drug captopril was approved in the US by the FDA in 1981 and came from the South American pit viper *Bothrops jararaca* [28]. Later, Sérgio Ferreira and colleagues discovered a set of nine peptides in the *Bothrops jararaca* venom that potentiated the bradykinin effect, which inhibits the angiotensin-converting enzyme (ACE). Subsequently, many other ACE inhibitors based on bradykinin potentiating factors (BPFs) were synthesized into Captopril, such as lisinopril, quinapril, and ramipril [27]. In the same sense, antiplatelet drugs like Tirofiban and Eptifibatide (approval for FDA and EMA) were synthesized based on Echistatin and Barbourin disintegrins from *Echis carinatus* and *Sistrurus miliarius barbourin* snake venom, respectively. Echistatin competes with fibrinogen (FG) for binding to the α_IIB_β_3_ integrin through an Arg-Gly-Asp (RGD) motif, which inhibits the final step in platelet aggregation [29]. Instead, Barbourin inhibits the glycoprotein IIb/IIIa through a Lys-Gly-Asp (KGD) motif [30]. Besides, in preclinical studies, we found the antithrombotic drug Anfibatide, an anticoagulant C-type lectin from the *Deinagkistrodon acutus* snake venom [31].

On the other hand, the Crotoxin (CTX), the main toxin from *Crotalus durissus terrificus* rattlesnake venom, was used in clinical trials with advanced cancer patients. A phase I clinical trial was developed on patients with advanced solid tumors using intramuscular injection for 30 successive days at amounts ranging from 0.03 to 0.22 mg/m^2^. Despite neurotoxicity being identified as a principal side effect, it was controllable in the study, and an effective anti-tumor activity was found in three patients with a > 50% tumor mass reduction. The clinical trial results displayed the CTX as a possible anticancer agent that could be used as a prototype under a Phase II clinical trial [32]. Later, a clinical trial aimed to demonstrate if intravenous injection of CTX in humans could be tolerated and reach more elevated and therapeutically effective dose levels without displaying the adverse effects associated with the intramuscular administration of this toxin. In this trial, neuromuscular toxicity was reported in up to 75% of patients. However, they propose intrapatient dose escalation to reduce the adverse side effects [33].

Therefore, as mentioned above, snake venoms constitute tools with pharmacological potential against diseases, including cancer. Thus, we described below the bioprospecting studies using snake venom and some purified proteins from these animals as new strategies against GB.

## Methods

**Figure 1.**
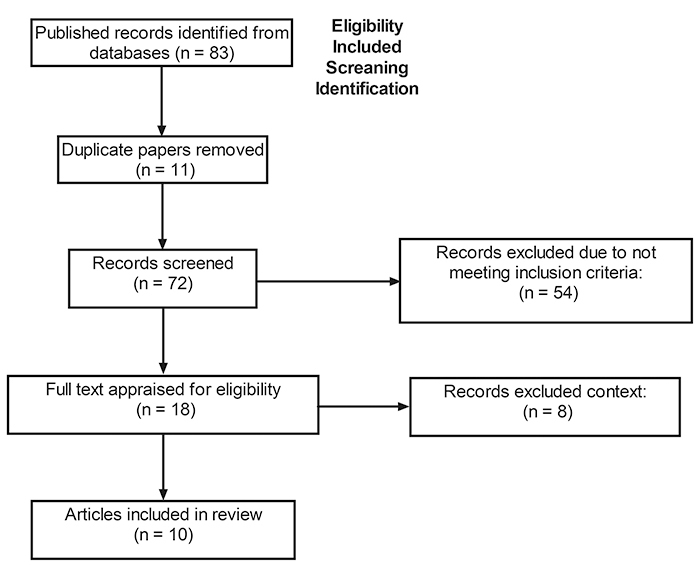
PRISMA flowchart showing the study design process.

In January 2024, a systematic search was performed in the PubMed, EMBASE, and Web of Science databases from January 2000 to December 2023 based on the PRISMA guidelines. *In vivo* and *in vitro* studies were selected, mandatorily conducted with Viperidae venom, and focused on GB. Search terms were selected based on the PEO framework and combined using Boolean operators ("AND", "OR"). The search strategy used the following descriptors according to Medical Subject Headings (MeSH): on Medline/Pubmed and Google Scholar, "Glioblastoma" or "glioma" or "high-grade glioma" or "WHO IV glioma" or "brain cancer" and "snake venom" or "Viperidae" or "bioprospection". The inclusion criteria to include a paper in our systematic review were that authors used whole venom, proteins, or peptides from Viperidae snake venom in *in vivo* or *in vitro* studies with glioblastoma cell lines or primary cell cultures. Besides, the authors included the methodology employed in each assay. The selection of the papers was performed in a standardized manner by two authors independently. A third author analyzed possible discrepancies. We identified 83 articles, of which 65 were excluded from the study after reading the title and abstract. A total of 18 articles were selected for full-text evaluation. The final inclusion, therefore, comprised 10 studies that met the proposed inclusion criteria and were used to construct this review. The PRISMA diagram is shown in Figure 1.

## Results

### In vitro effects of Viperidae venoms on GB cells

Regarding the research interest in proving the usefulness of viperid venoms as molecule sources to generate a directed action on glioblastoma, several investigations have determined a potential action of different venoms on these tumor cells - for example, Soares et al. [34] evaluated the *Crotalus durissus terrificus* snake venom and the main polypeptide, CTX, against RT2 glioma cells. CTX is a Beta-neurotoxin composed heterodimerically by an acidic protein and a basic protein with phospholipase A_2_ activity. For some PLA_2_s, the cytotoxic activity on cancer cells is independent of its PLA_2_ catalytic activity. The selective effect on tumor cells is probably related to the interaction through its C-terminal region with molecules of cytoplasmic membranes, such as integrins [35-40]. The snake venom and CTX displayed morphological changes in the cellular shape of RT2 glioma cells, from cell shrinkage to bleb formation. In addition, snake venom and CTX showed nuclear condensation, DNA fragmentation, and perinuclear apoptotic body formation at the nuclear level on RT2 glioma cells. When performing cell cycle analysis by flow cytometry, they found that snake venom increased the subG1 cell population, suggesting cell cycle arrest, probably in an attempt to repair the damage induced by the snake venom. However, despite the result obtained by the two treatments, the CTX at 100 μg/mL was cytotoxic only to 11 ± 0.57% (IC_50_ > 100 μg/mL) compared with the IC_50_ of the whole venom = 2.15 ± 0.20 μg/mL. This result established that snake venom displayed high cytotoxicity on the brain tumor cells with low sample quantity due to synergism between the different protein family combinations that venom contains [34]. 

Additionally, Nalbantsoy et al. [6] evaluated the cytotoxic effect of the complete venom of two viper species (*Montivipera raddei* and *Montivipera bulgardaghica*) on several cell lines, including U87MG glioblastoma. Both venoms showed concentration-dependent cytotoxicity against this cell line, with an IC_50_ of 6.04 ± 0.47 and 1.25 ± 0.38 mg/mL for the *M. bulgardaghica* venom and *Montivipera raddei* venom, respectively. However, the most significant cytotoxic effect was found in the A-549 human alveolar adenocarcinoma cell line, so the subsequent assays were carried out on this cell line.

Finally, Ozverel et al. [41] evaluated the whole snake venom cytotoxicity from *Cerastes cerastes* and *Cryptelytrops purpureomaculatus* against several tumorigenic cell lines, including the GB astrocytoma cell line U87MG. After 48 h of U87MG treatment with the different snake venom concentrations, the *C. cerastes* snake venom displayed the most cytotoxicity activity (IC_50_ = 0.88 ± 0.44μg/mL) compared with the *C. purpureomaculatus* venom (IC_50_ = 1.32 ± 0.03μg/mL). However, despite the results, the cytotoxicity of snake venom on non-tumorigenic cell line - HEK293 showed a low IC_50_ (2.36 ± 1.04 and 2.04 ± 0.21μg/mL to *C. cerastes* and *C. purpureomaculatus*, respectively).

**Figure 2.**
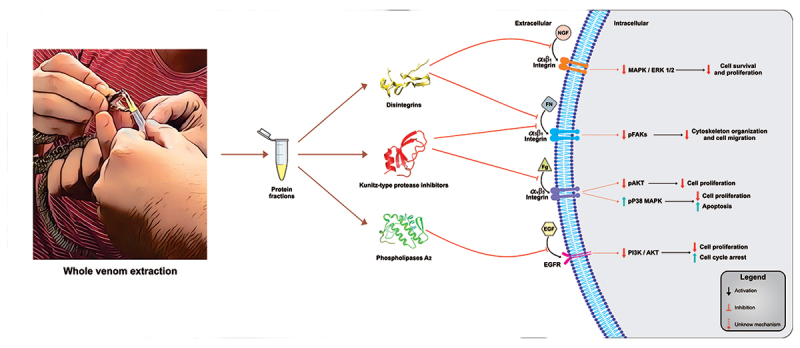
The possible interaction of snake venom proteins (for example Disintegrin, PDB code: 8S9E; Kunitz-type protease inhibitors, PDB code: 1JC6; Phospholipase A_2_, PDB code: 1Q5T) with transmembrane receptors of GB cells affects intracellular pathways. Abbreviations: NGF (Nerve growth factor), FN (Fibronectin), FG (Fibrinogen), and EGF (Epidermal growth factor).

Although the reviewed works present significant effects for viperid venoms against GB tumors, the approaches with whole venoms have some things that could be improved. Most importantly, in these studies, no evaluations demonstrate innocuousness for the venoms evaluated, so it is impossible to rule out that the venom is non-specific against non-tumorigenic cells. Another area for improvement is that working with whole venom implies that effects are evaluated from a cocktail of molecules, so it is impossible to know which molecule or molecules are mainly responsible for the evidenced effect. However, it is necessary to highlight that one of the works does isolate the molecule (CTX) that the authors hypothesized was mainly responsible for the cytotoxic action evidenced by the complete venom, and experiments were carried out to validate or rule out the hypothesis. Regarding the other reviewed publications, we hope that in the following author investigations, they try to identify the proteins that produce the cytotoxicity promissory effect on GB cell lines. The antitumor effects of the whole venom mentioned above on GB cells are summarized in Table 1 and Figure 2, respectively.

### Effects of isolated proteins on GB cancer cells

Disintegrins, phospholipase A_2_ (described above), and Kunitz-type serine protease inhibitors are the snake venom proteins from the Viperidae family that have been used with a possible anticancer effect against GB. Disintegrins are small (MW 7-10 kDa), non-toxic, non-enzymatic, and cysteine-rich proteins that bind to different integrin receptors responsible for cell adhesion [42]. They showed a structure with arginine-glycine-aspartic acid (RGD) or non-RGD (such as MLD, KTS, ECD, KGD, VGD, RTS, WGD) motif that binds to integrin-type receptors inhibiting the proliferation of cancer cells, angiogenesis, and triggering apoptosis [43-45]. Kunitz-type protease inhibitors are small proteins found in Elapidae and Viperidae snake venoms, consisting of a monomeric chain containing about 60 amino acids with three disulfide bridges [46, 47]. Functionally, it exhibits a potent antitumor cell effect inhibiting cell adhesion, migration, and invasion. This protein exerted these effects by interfering with α_v_β_3_ integrin through an RGD-like motif (^41^RGN^43^) modulating PI3K/AKT (Phosphoinositide 3-kinase/ Protein kinase B) and MAPK (Mitogen-activated protein kinase) signaling pathways [48]. The antitumoral effects of proteins described above on GB cell lines are detailed in the following lines.

Brown et al. [49] employed a disintegrin antagonist of the integrin α_9_β_1_, VLO5, from *Vipera lebetina obtusa* snake venom on the LN229 cell line (overexpressing α_9_β_1_). They evaluate if VLO5 affects the normal interaction between the nerve growth factor (NGF) and α_9_β_1_ integrin, related to activation of MAPK Erk1/2 pathway and subsequent migration and proliferation of GB cells. These experiments showed that VLO5 interacts with α_9_β_1_ integrin, prevented/inhibited binding with NGF, affecting proliferation, and activating the caspase 9-dependent apoptosis pathway (intrinsic pathway) on the LN229 cell line [49]. These results are exciting in finding a molecule with the potential to attack GB cells selectively, in this case, those that express the α_9_β_1_ integrin, which is not expressed in non-tumorogenic brain tissue. However, it should be considered that a drug with this approach would not be a complete solution for the selective targeting of GB cells since there are also cells in the tumor microenvironment that do not express the α_9_β_1_ integrin.

On the other hand, in the GB metastatic spread, the invasion of adjacent tissue is a complex process that involves different steps, such as adhesion, rupture of the extracellular matrix, and tumoral locomotion mechanisms. These processes involve the activity of serine proteinases, endoglycosidases, and matrix metalloproteinases (MMPs), among others. Besides, the integrins-specific association of α_v_β_5_ and α_v_β_3_ to metalloprotein family members has been reported to play an essential function in the potential of tumor endothelial cell and migration regulation. Therefore, Schmitmeier et al. [50] wanted to evaluate if the effect on invasion and migration in GB was associated with the integrins and mediated by extracellular matrix degradation. For this purpose, they performed zymography assays using a lysate of different GB cultures (cell lines displaying α_5_β_1_ and α_v_β_5_ expressions: T98G, U87MG, A-172, and U138) that were previously treated with the disintegrin contortrostatin (CN) purified from *Agkistrodon contortrix contortrix* snake venom. They measured the MMP-2, MMP-9, and plasminogen activator (PA)/Plasmin system activity and their natural tissue inhibitor of metalloproteinases (TIMP), TIMP-1 and TIMP-2. There were no differences in activity in any of these molecules when comparing cells treated with CN and untreated cells. Therefore, they concluded that inhibition of spread and migration was not associated with tumor proteolytic activity. Finally, in the same investigation, they found that CN inhibits the migration of tumor cells, concluding that invasion inhibition occurs by alteration in locomotion rather than proteolytic activity [50].

Later, Schmitmeier et al. [51] evaluated the signaling pathways activated after the binding of CN to integrin α_5_β_1_, contrasting the effect in cell signaling without the CN interference to fibronectin - FN (physiological ligand of integrin α_5_β_1_). They found a greater affinity of integrins for CN than FN, displacing it from its receptor and decreasing signaling. Furthermore, CN binding alters the signaling pathways involved in the integrin α_5_β_1_/FN interaction, decreasing the phosphorylation levels of focal adhesion kinases (FAKs), including Src and paxillin. Besides, when CN was added to the tumoral cell lines A-172 and U87MG preincubated with FN, detachment and morphological changes like a retraction of cell extensions, formation of knob-like tails, and cell rounding were observed. These effects were associated with changes in the actin cytoskeleton and a migration decrease [51].

On the other hand, Morjen et al. [46] worked with a Kunitz-type serine protease inhibitor, PIVL, characterized by *Macrovipera lebetina transmediterranea* snake venom. They evidenced affection for cell adhesion, migration, invasion ability, and motility on the U87MG GB cell line. Furthermore, they related these effects to the cells' interaction disruption with the extracellular matrix proteins FG and FN. Later, they showed on the HMEC-1 cell line that the inhibitory effect on cell adhesion and migration induced by PIVL is related to the blocking of integrin interactions with the extracellular matrix proteins postulated in the previous study, explicitly blocking the interactions between α_5_β_1_/FN and α_v_β_3_/FG [48]. In a posterior investigation, Morjen et al. [52] also produced a recombinant protein variant of native PIVL (PIVL) called rPIVL. They demonstrated that rPIVL efficiently inhibited the adhesion of U87MG in a concentration-dependent manner. Besides, rPIVL significantly reduced the neovascular density on the U87MG GB cell line, and the PI3K/AKT and MAPK signaling pathways were affected. The antitumor effects of the proteins mentioned above on GB cells are summarized in Table 1 and Figure 2, respectively. 

**Table 1.** Principal effects of the whole venom and proteins from Viperidae snake venom against GB cell lines.

Species	Venom/Protein	Protein Family	Cell line	Principal effects in GB cells	Reference
*Cerastes cerastes*	Whole venom	N/A	U87MG	Cytotoxicity	[41]
*Cryptelytrops purpureomaculatus*	Whole venom	N/A	U87MG	Cytotoxicity	[41]
*Crotalus durissus terrificus*	Whole venom	N/A	Rat glioma RT2	Dose and time-dependent cytotoxicity Increase of subG1 cells Morphological alterations characteristics of apoptosis	[34]
	Crotoxin	Phospholipase A_2_		Morphological alterations characteristics of apoptosis	
*Agkistrodon contortrix contortrix*	Contortrostatin	Disintegrin	A-172 and U87MG	It does not cause cell death in both glioma cell lines Induces phosphorylation of focal adhesion kinases (Paxillin and p130Cas) in both cell lines Higher binding affinity for integrin receptors than fibronectin in both cell lines Migration inhibition in both cell lines	[51]
	Contortrostatin	Disintegrin	T98G, U87MG, A-172, and U138	No interference with the viability of glioma cells Interference with adhesion to vitronectin and fibronectin but not with laminin and collagen on glioma cells Inhibits glioma cell invasiveness in all cell lines, although less pronounced in U138	[50]
*Vipera lebetina obtusa*	VLO4 and VLO5	Disintegrin	LN229 and LN18	Proliferation inhibition, apoptosis induction, and angiogenesis reduction on LN229 cell line (positive for α9β1expression) No effect in cell proliferation on LN18 (negative for α9β1expression)	[49]
*Macrovipera lebetina transmediterranea*	PIVL (Kunitz-type protease inhibitor)	Serine Protease inhibitor	U87MG	Migration inhibition Invasion reduction in a concentration-dependent manner	[46]
	rPIVL (Kunitz-type protease inhibitor)	Serine Protease inhibitor (Recombinant Protein)		Decreased neovascular density Reduced expression of phosphorylated AKT Increased expression of phosphorylated P38 MAPK	[52]

N/A: Not applicable

### *In vivo* antitumoral effect of snake venom isolated proteins against GB

Due to the antitumor results obtained *in vitro* with proteins isolated from snake venoms and GB cell lines, recombinant Vicrostatin (VCN) was synthesized based on the CN amino acid sequence. The VCN, a disintegrin with a molecular weight of 7146 Da, was designed with a recombinant technology adding a carboxy-terminal extension of CN, increasing the binding affinity for integrin α_5_β_1_, a transmembranal receptor associated with angiogenesis [53]. Later, the VCN effects were evaluated in two *in vivo* models, as described below.

Bose et al. [54] evaluated the effect of VCN on GB growth and angiogenesis using athymic nu/nu mice with a U87MG cell line implanted into the subcutaneous space of the right dorsal flank. No adverse effects were evidenced after delivering VCN into the blood system, and mice tolerated VCN throughout the 28-day trial. On average, the tumor for VCN treatment was reduced by 46.2%, and the microvasculature density decreased six times.

On the other hand, Swenson et al. [53] evaluated the anticancer *in vivo* efficacy of VCN bound to the beta-emitting radionuclides ^131^I, called ^131^I-VCN. This conjugated molecule was used to assess the prolonging of progression-free survival in two distinct xenograft glioma models lacking O-6-methylguanine methyltransferase expression (U87MG and U251 cell lines) and, therefore, sensitive to TMZ. To perform an orthotopic xenograft model, tumors were implanted in mice brains using stereotactic injections. There were five study groups: negative control, ^131^I control, ^131^I-VCN treatment, external beam radiotherapy (EBRT) plus TMZ, and combined ^131^I-VCN plus TMZ therapy. The mice treated with ^131^I-VCN did not show adverse effects. ^131^I-VCN and ^131^I-VCN + TMZ groups displayed a significant therapeutic advantage over the control, while the ^131^I group exhibited no significant therapeutic effect. In addition, the ^131^I-VCN + TMZ treatment combination showed better efficacy than the standard of care EBRT/TMZ combination [53]. These results could indicate that VCN, as a precision delivery system of targeted radioactivity, exerts a synergistic effect with TMZ, generating a strategy to deal with some patients' resistance to TMZ. Additionally, this study is extremely valuable in terms of knowledge about the ability of snake venom proteins to cross the blood-brain barrier and interact with GB cells since it broadens the possibilities for considering these molecules as a basis for drug design with the potential for a selective and directed attack against brain tumor cells.

In addition to murine biomodels, another type of biomodel has been used to determine the antitumor potential of molecules isolated from viperids on glioblastoma: the chick embryo chorioallantoic membrane (CAM). Three proteins discussed above (VLO5, PIVL, and rPIVL) were used to determine antiangiogenic potential in a shell-less quail egg assay in which GB cells were implanted on top of CAM. Tumor growth (evaluated by the spreading area of LN229 implanted cells), tumor weight, and vascularization of the entire CAM significantly decreased after VLO5 treatment, in contrast to NGF treatment [49]. For PIVL, an antiangiogenic effect on CAM was also evidenced: the total vessel length was reduced by 61% compared to the control. Interestingly, this effect was the same when using a synthetic RGN peptide based on the PIVL sequence, confirming that the antiangiogenic effect of PIVL is mediated by its RGD-like motif [48]. On the other hand, the recombinant protein of PIVL (rPIVL) showed a similar reduction effect on total vessel length (55%) [52]. The antitumor effects of the proteins mentioned above on the GB in biomodels are summarized in Table 2.

**Table 2.** Principal *in vivo* effects of proteins isolated from snake venom of the Viperidae family against glioblastoma.

Snake	Cell line	Agent	Biomodels	Site tumor induction	Administration mode	Principal effects in glioblastoma tumor cells	Reference
*Agkistrodon contortrix contortrix*	U87MG	Vitronectin (VN)	Athymic *nu/nu* mice (Four to six weeks old)	Subcutaneous space of each nude mouse’s dorsal right flank	Drugs were delivered directly into the blood system by tail vein injections	The microvessel density was bigger on the control group tissues than on the VN-treated group tissues The control group had approximately 5.6 times more microvessel density than the tumors in the VN-treated group	[54]
	U251 human glioma cell	^131^I-VCN	Balb/c *nu/nu* mice (Five weeks old)	Tumors were implanted 3 mm deep in the midline of mice brains using stereotactic injections	Intravenously	^131^I-VCN plus TMZ significantly enhances the mice survival treated more than ^131^I-VCN alone	[53]
*Vipera lebetina obtusa*	LN229	VLO5	Chick chorioallantoic membrane (CAM)	N/A	N/A	Tumor growth, weight, and vascularization significantly decreased after VLO5 treatment	[49]
*Macrovipera lebetina transmediterranea*	U87MG	PIVL	Chick chorioallantoic membrane (CAM)	N/A	N/A	Total vessel length was reduced by 61% compared to the control	[46]
		rPIVL		N/A	N/A	Total vessel length was reduced by 55% compared to the control	[52]

N/A. Not applicable

### Snake venom proteins and blood-brain barrier

Most drugs that cross the blood brain barrier (BBB) can do it by transmembrane diffusion (TD) [55]. TD is a non-saturable mechanism that depends on the drug melding into the cell membrane. Low molecular weight and high lipid solubility favor crossing by this mechanism. However, other molecular properties like the charge, tertiary structure, and degree of protein binding are additional factors that affect the ability of a drug to cross the BBB [56]. For example, as described above, the modification generated to VCN induced a fold close to the RGD-containing disintegrin loop, increasing the interaction with the integrin α_5_β_1_ receptor and helping to overpass the blood-brain barrier. Moreover, the high density of integrin targets expressed by the glioma neovasculature supports the VCN's ability to penetrate the BBB [53]. For that reason, the design of new drugs could consider the high receptor expression of the pericytes, such as integrins, to facilitate the cross BBB and reach the tumor microenvironment.

## Conclusion

Clinical GB management has not achieved significant changes in the last 20 years. However, the standard of care continues to be surgery with maximum safe resection, chemotherapy, and radiotherapy. All clinical trials mentioned in this review compared with the Stupp protocol impact disease-free progression without changes in overall survival. However, numerous ongoing clinical trials approach the need to search for new alternatives that increase survival and reduce the adverse effects of current therapy. In this sense, immunotherapy and target therapies emerge as promising alternatives, although still without results in GB.

Thus, there are an increasing number of bioprospecting studies, among which are developed with molecules from snake venom. This review showed *in vitro* and *in vivo* studies with GB models with an antitumoral effect of the whole snake venom from the Viperidae family or its fractions (PLA_2_, disintegrins, and serine proteases inhibitors). This antitumoral effect includes cell cycle alteration, cell death by intrinsic apoptosis induction, inhibition of cancer cells' metastatic ability (cell adhesion, migration, invasion, and angiogenesis), reduction of tumoral growth, and synergic effect with a chemotherapeutic agent (TMZ). These findings provide new perspectives for GB treatment and can be used to design effective drugs. In this sense, studies in bioprospecting exploring the advantage of snake venom proteins against GB deserve a chance to be explored due to their high specificity, small size, inherent bioactivity, and few side effects to cross the BBB reach the tumor microenvironment.

### Abbreviations

ACE: Angiotensin-converting enzyme; AKT: Protein kinase B; BBB: Blood-brain barrier; BPFs: Bradykinin potentiating factors; BRAFV600E: v-raf murine sarcoma viral oncogene homolog B1; BVZ: Bevacizumab; CAM: Chick chorioallantoic membrane; CAR-T: T cells with chimeric antigen receptors; CDK: Cyclin-dependent kinases; CD80: Cluster of differentiation 80; CN: Contortrostatin; CTLA-4: Cytotoxic T-Lymphocyte Antigen 4; CTX: Crotoxin; DIS: Disintegrin; EBRT: external beam radiotherapy; EGF: Epidermal growth factor; EGFR: Epidermal growth factor receptor; EGFRvIII: Epidermal growth factor receptor variant III; FAK: Focal adhesion kinases; FG: Fibrinogen; FN: Fibronectin; GB: Glioblastoma; IDH: Isocitrate dehydrogenase; KGD: Lys-Gly-Asp; MMP-2: Metalloproteinase-2; MMP-9:Metalloproteinase-9; NGF: Nerve growth factor; NTRK: Neurotrophic tyrosine kinase-1 receptor; OS: Overall survival; PA: Plasminogen activator; PD-L1: Programmed cell death-1 ligand; PD-L1/2: Programmed Death-ligand ½; PD-1Programmed cell death protein 1; PEO: Population/exposure/outcome; PFS: Progression-free survival; PI3K: Phosphoinositide 3-kinases; PIVL: Kunitz-type protease inhibitor; PLA_2_: Phospolipase A_2_; PRISMA: Preferred Reporting Items for Systematic Reviews and Meta-Analysis; RAS: Rat sarcoma virus protein/ Phosphoinositide 3-kinases; RGD: Arg-Gly-Asp; rPIVL: Recombinant protein of PIVL; TD: Transmembrane diffusion; TAMs: Tumor-associated microglia and macrophages; TIMP: Tissue inhibitor of metalloproteinases; TME: Tumor microenvironment; TMZ: Temozolomide; VEGF: Vascular endothelial growth factor; VCN: Vicrostatin; VN: Vitronectin.

## Data Availability

Not applicable.
